# Recovery of memory retention after anesthesia with remimazolam: an exploratory, randomized, open, propofol-controlled, single-center clinical trial

**DOI:** 10.1186/s40981-023-00635-7

**Published:** 2023-07-13

**Authors:** Keiko Nobukuni, Kazuhiro Shirozu, Aiko Maeda, Kouta Funakoshi, Midoriko Higashi, Ken Yamaura

**Affiliations:** 1 Operating Rooms, Kyushu University Hospital, Fukuoka, Japan; 2 Department of Anesthesiology and Critical Care Medicine, Kyushu University Hospital, Fukuoka, Japan; 3 Center for Clinical and Translational Research, Kyushu University Hospital, Fukuoka, Japan; 4 Department of Anesthesiology and Critical Care Medicine, Graduate School of Medical Sciences, Kyushu University, Fukuoka, Japan

**Keywords:** Anesthesia, Memory, Propofol, RCT, Remimazolam, Amnesia

## Abstract

**Purpose:**

Remimazolam, a newly developed ultra-short-acting benzodiazepine, provides early recovery of consciousness but its effects on memory recovery are unclear. This study examined memory recovery after emergence from general anesthesia using remimazolam.

**Methods:**

Seventy-four patients undergoing breast surgery between October 2021 and March 2022 were enrolled and randomly assigned to receive propofol (control group) or remimazolam as general anesthetic during surgery. The primary endpoint was the number of posters patients remembered 24 h after surgery (among four posters shown after recovering from anesthesia) as an assessment of memory retention. The secondary endpoints were the recall of a numeric character patients had been shown just before anesthetic induction, as an assessment of retrograde amnesia 24 h after surgery.

**Results:**

Sixty-six patients (propofol, 32; remimazolam, 34) were assessed. Patients in the remimazolam group remembered significantly fewer posters shown to them after surgery than those in the propofol group (0 [0 − 2] vs. 2 [1 − 3], *p* < 0.001). In the remimazolam group, the patients who received flumazenil remembered a higher number of posters than those who did not receive flumazenil (3 [1 − 4] vs. 0 [0 − 0], *p* < 0.001). All patients remembered all events that occurred during the preoperative period as well as the numeric character.

**Conclusion:**

Patients recovering from remimazolam anesthesia without receiving flumazenil do not remember events after regaining consciousness.

**IRB:**

Kyushu University School of Medicine Hospital Institutional Review Board (IRB) (approval number: 20212006).

**Trial registration:**

This clinical trial was registered with the University Hospital Medical Information Network (UMIN) Center on September 28, 2021 (UMIN-CTR: UMIN000045593).

**Implication statement:**

Memory recovery is slower following emergence from remimazolam than from propofol anesthesia.

**Supplementary Information:**

The online version contains supplementary material available at 10.1186/s40981-023-00635-7.

## Introduction

Remimazolam, a newly developed ultra-short-acting benzodiazepine, differs from midazolam in that its metabolites have no pharmacological sedative effects and that its effects disappear rapidly upon the discontinuation of administration [1]. Benzodiazepines including midazolam affect memory retention and lead, for example, to antegrade amnesia [2–4]. It has been demonstrated that the impairments induced by benzodiazepines concern the acquisition of information [5]. On the other hand, antegrade amnesia effects of anesthetics are beneficial, improve patient comfort, and can be used in various clinical situations. Our previous study suggested that remimazolam might cause delays in memory recovery [6]; however, the details underlying this observation remain unclear. We hypothesized that although anesthesia with remimazolam would lead to early recovery of consciousness, there would be no memory after awakening and memory recovery would be delayed.

This study thus aimed to examine the effects of general anesthesia using remimazolam on memory recovery.

## Materials and methods

This study was an exploratory, open, propofol-controlled, single-center, randomized clinical trial (RCT) set up to compare memory recovery after general anesthesia with either remimazolam or propofol. The study design was approved by the Kyushu University School of Medicine Hospital Institutional Review Board (IRB) (approval number: 20212006) and was conducted in accordance with the World Medical Association Declaration of Helsinki. The study was also registered in the UMIN Clinical Trials Registry (UMIN-CTR: UMIN000045593, September 28, 2021).

Patients scheduled to undergo breast surgery under general anesthesia were assessed for eligibility. These criteria were chosen to ensure uniformity of intraoperative surgical techniques. To prevent differences in cognitive functions, only patients between 20 and 65 years of age were included. The exclusion criteria were a history of hypersensitivity to remimazolam or propofol, egg or soybean oil allergies, acute angle-closure glaucoma, myasthenia gravis, serious disease complications, an American Society of Anesthesiologists (ASA) physical status of ≥ IV, shock, or coma resulting in cognitive function impairments. These criteria remained constant throughout the study.

After obtaining written informed consent, participants were randomly assigned to two groups using stratified block randomization. An allocation supervisor appointed by the Data Center of the Clinical Research Promotion Department of the Clinical and Translational Center at our institution prepared an allocation table intended to avoid clinical involvement in the trial. The discontinuation criteria and the guidelines for monitoring adverse events have been described in detail in an earlier protocol [7].

### Randomization

The electronic data capture software used in this study referred to the separately prepared allocation table, applying an allocation ratio of 1:1. Participants were randomly assigned following permuted block randomization using random block sizes of 2 and 4.

### Anesthesia method

Remimazolam was initiated at 6 mg/kg/h, which was changed to 1 mg/kg/h after confirming that the patient was asleep. During surgery, the dose was adjusted as required in the range of 0.5–2 mg/kg/h with reference to the Bispectral Index (BIS) value. The initial concentration of propofol was 3 µg/mL at the target blood concentration, administered using a target-controlled infusion pump (Terufusion™, Terumo Corporation, Japan) and adjusted with reference to the BIS value. The target value of BIS was set to 40–60 for both groups.

If the patient was still unconscious 5 min after the end of the surgery, flumazenil was administered intravenously to antagonize remimazolam, slowly and at an initial dose of 0.2 mg. If wakefulness was not achieved within 4 min after administration, an additional 0.1 mg was administered. If necessary, 0.1 mg was repeatedly administered at 1-min intervals, up to a total dose of 1 mg.

For concomitant medications, the usual dosage of fentanyl and remifentanil for intraoperative analgesia was administered. The dosage of remifentanil did not exceed a maximum of 0.5 µg/kg/min.

### Interventions

Memory retention before and after general anesthesia was evaluated as follows. Prior to the induction of anesthesia, the patient was presented a single-digit numeric character. Consciousness after recovery from anesthesia was defined as the time a patient was able to say her name in the operating room, with the anesthesiologist repeatedly instructing her to do so following extubation. When consciousness was confirmed, memory recovery was assessed by determining whether the patient could retain new information. To that end, the patient was shown four laminated A4-size posters that contained easily recognizable simple images: a car, a dog, a banana, and an umbrella, and asked to identify each image (Supplement 1) [8].

### Measurements

Twenty-four hours after surgery a blinded investigator asked each patient two questions to assess memory retention. First, the investigator asked the patients if they remembered the numeric character shown to them before the induction of anesthesia, and recorded the number of events (among a total of five) the patient remembered from before the start of anesthesia (ambulatory entry, bed transfer, vein insertion, BIS monitor attachment to the forehead, and mask fitting) and whether the patient remembered being shown the posters before the induction of anesthesia. Second, the investigator asked the patients what posters they had been shown after they after they had been confirmed to have regained consciousness.

### Data collection

Data were recorded and stored using REDCap software (Nashville, TN, USA) while the study was in progress and then exported from the database into statistical analysis software [9, 10]. REDCap, which ensures higher levels of data privacy and security protection than other data storage platforms, has an audit trail that left a record every time a participant or staff member made changes to any data entered on the website. Data and outcome assessors were blinded.

### Sample size

No similar studies have been conducted in the past that could have served as a reference. We therefore set the number of patients in each of the two groups to 35, as we estimated that we would be able to enroll this many participants within approximately 6 months.

### Primary and secondary endpoint analysis

The primary endpoint was the memory recovery, determined by our assessment of memory retention, that is, the number of posters remembered after surgery. The secondary endpoint was the assessment of retrograde amnesia, determined by evaluating memory retention before anesthesia.

Because this small exploratory study aimed to provide basic data for appropriately powered large-scale RCTs for future investigations, we conducted a descriptive statistics per-protocol set analysis. The same full analysis set was applied as a sensitivity analysis of the primary endpoint, and all analyses were performed with a two-sided significance level of 0.1. To assess differences in the primary and secondary outcomes between the groups, data derived from continuous variables are presented as means (SD: standard deviations) or medians (IQR: interquartile ranges), and data derived from non-continuous variables are presented as numbers and percentages. Continuous variables were analyzed using the Mann–Whitney *U* test or Student’s *t* tests, and categorical variables were analyzed using chi-square tests.

## Results

### Baseline characteristics

**Fig. 1:**
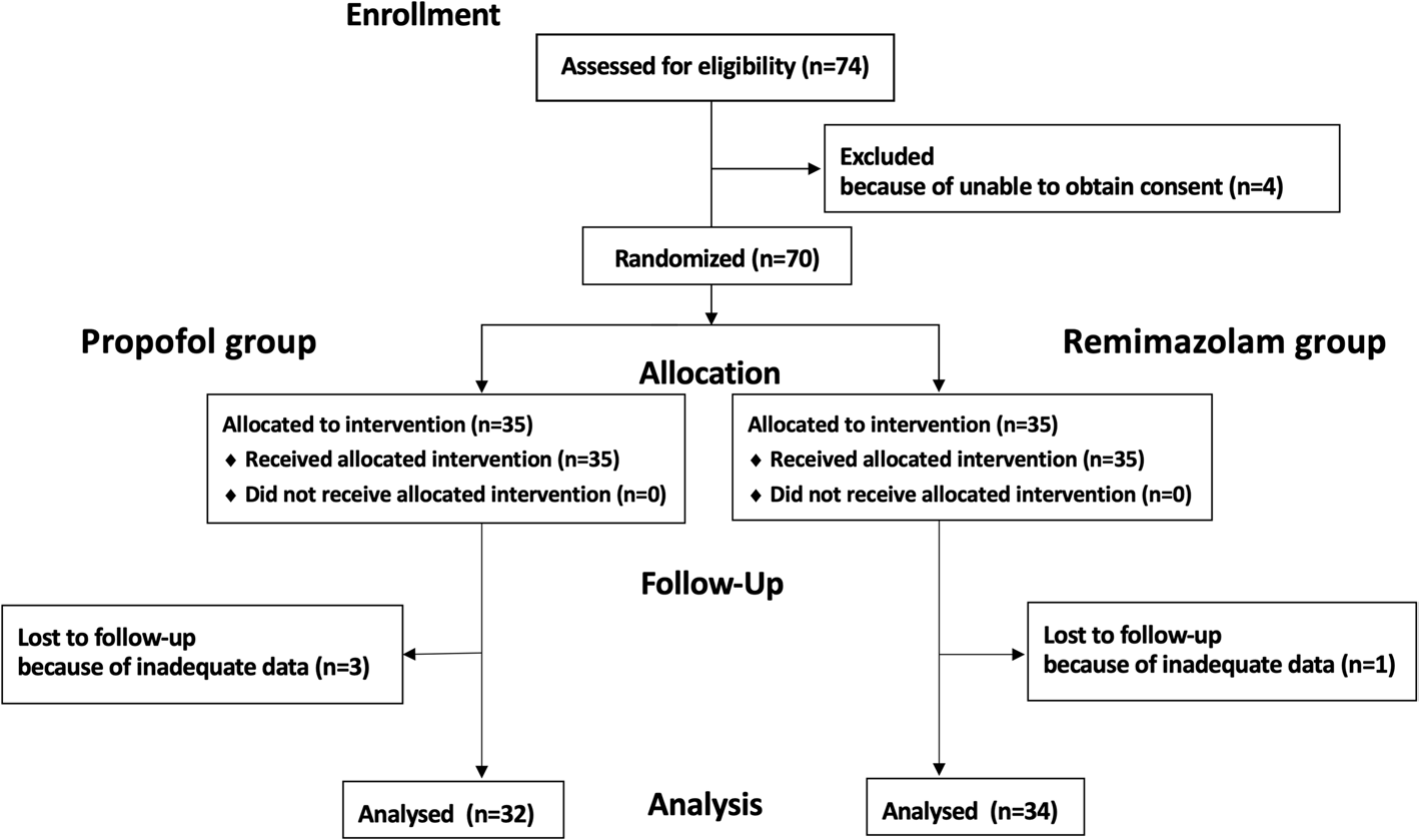
Flow diagram

In total, 74 patients treated at Kyushu University Hospital from October 20, 2021 to April 13, 2022 were assessed in this study; four patients were excluded before randomization because consent could not be obtained. A total of three patients in the control group and one in the remimazolam group were excluded because some of their data at operation room (OR) entry were missing. Finally, the data of 66 patients, 32 in the control and 34 in the remimazolam group, were analyzed (Fig. 1). No data on memory retention were missing in the full-analysis set; therefore, no value imputation was required. The two groups did not differ in basic preoperative characteristics (Table 1).

**Table 1:** Pre- and intraoperative characteristics

	Propofol group (*n* = 32)		Remimazolam group (*n* = 34)		*p* value
	Mean	SD	Mean	SD	
Preoperative characteristic values					
Age (years)	50.4	7.6	51.1	10.0	
Height (cm)	157.1	5.7	158.0	4.6	
Weight (kg)	55.3	10.5	58.7	10.9	
Hypertension (%)	12.5		11.8		
Dyslipidemia (%)	9.4		26.5		
Diabetes mellitus (%)	3.1		5.9		
Cerebrovascular disease (%)	0.0		0.0		
Oral hypnotic drugs on the day of surgery (%)	6.3		17.6		
Intraoperative characteristic values					
Operating time (min)	113	48	118	39	0.69
Anesthesia time (min)	163	55	169	44	0.62
Total dose of fentanyl (µg)	215.6	49.9	216.2	79.5	0.97
BIS value before anesthesia	98	1	98	1	0.23
BIS value during surgery (mean)	46	6	54	9	< 0.001
BIS value before leaving OR (the time at which the patients responded)	95	4	88	8	< 0.001

*BIS* bispectral index, *OR* operating room, *SD* standard deviation

### Intraoperative characteristics

**Fig. 2:**
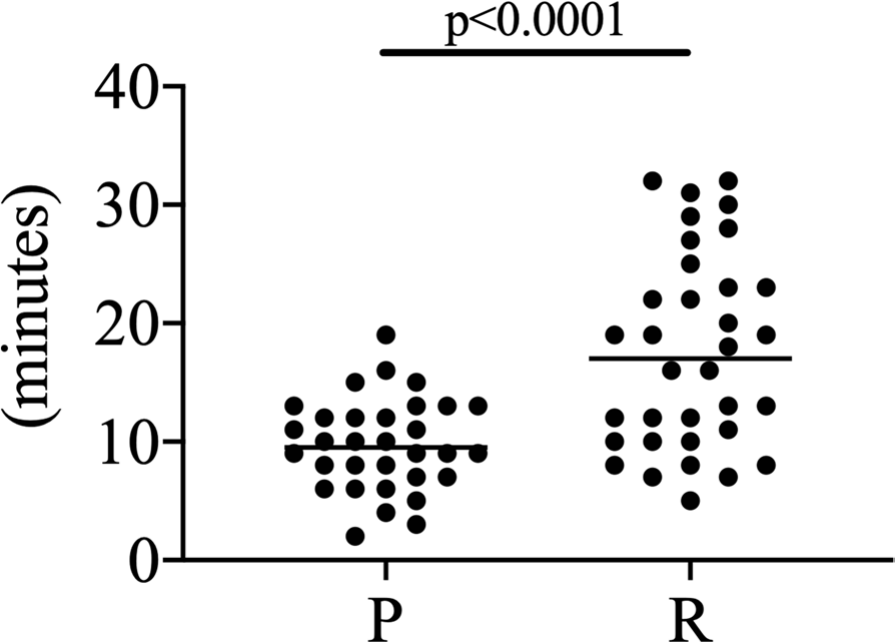
The elapse time from terminating the infusion of the anesthetic agents. P: propofol group, R: remimazolam group

The mean BIS values during surgery were significantly higher in the remimazolam group than those in the propofol group (Table 1). The elapsed time from terminating anesthetics to extubation was significantly longer in the remimazolam group than that in the propofol group (17.6 ± 8.3 min. vs. 9.7 ± 3.9 min., *p* < 0.0001) (Fig. 2). The mean BIS values before leaving the OR were significantly lower in the remimazolam group than those in the propofol group (Table 1).

### Memory retention

All patients in both groups remembered the numeric character shown to them before the induction of anesthesia and the five events that occurred before the start of anesthesia. The patients in the remimazolam group remembered fewer posters shown to them after surgery than those in the propofol group (0 [0 − 2] vs. 2 [1 − 3], *p* < 0.001) (Table 2).

**Table 2:** Comparison of primary and secondary outcomes between the propofol and remimazolam group

	Propofol group (*n* = 32)		Remimazolam group (*n* = 34)		*p* value
	Median	IQR	Median	IQR	
Primary outcome: anterograde amnesia					
Posters shown just before leaving the OR (number)	2	1–3	0	0–2	< 0.001
Secondary outcome: retrograde amnesia					
Numeric character shown just before induction	100%		100%		
Events occurring before induction (number)	5	5–5	5	5–5	

*IQR* interquartile ranges, *OR* operating room

### The effect of flumazenil: subgroup analysis

Flumazenil was administered to 10 patients in the remimazolam group. The mean BIS value before leaving OR did not differ significantly between the patients who did and did not receive flumazenil. The patients who received flumazenil remembered a higher number of posters shown to them after surgery than those who did not receive flumazenil (3 [1 − 4] vs. 0 [0 − 0], *p* < 0.001) (Table 3). The patients in the remimazolam group who did not receive flumazenil remembered fewer postoperative posters than those in the propofol group (0 [0 − 0] vs. 2 [1 − 3], *p* < 0.0001).

**Table 3:** Add-on attenuation effect of flumazenil on amnesia in the remimazolam group

Flumazenil	( +) (*n* = 10)		( −) (*n* = 24)		
	Mean	SD	Mean	SD	*p* value
BIS value before leaving OR (the time at which the patients responded)	92	8	87	8	0.19
	Median	IQR	median	IQR	*p* value
Posters shown just before leaving the OR (number)	3	1–4	0	0–0	< 0.001

*BIS* bispectral index, *IQR* interquartile ranges, *OR* operating room

## Discussion

The results of this study show that memory before anesthetic induction was retained with both remimazolam and propofol while memory after general anesthesia was retained only with propofol.

Our finding that memory before anesthetic induction with remimazolam or propofol was retained indicates that patients did not have retrograde amnesia. This is consistent with previous reports [4, 11, 12].

The fact that memory after general anesthesia was retained with propofol but not with remimazolam suggests that the processes underlying the recovery of consciousness and memory differ in patients receiving one or the other drug. We defined recovery of consciousness as the time point when the patient was able to recall their name after emerging from general anesthesia; thus, we examined memory retention at the same level of consciousness after general anesthesia with remimazolam or propofol. The mechanism leading to poorer memory recovery following general anesthesia with remimazolam is not clear, but it may be related to residual anesthetics. A previous report that described the moderate and short-lasting residual effect of remimazolam after 2 h of conscious sedation also indicated the existence of residual remimazolam at showing picture to patients in this study [13]. A sub-analysis of the remimazolam group—with or without flumazenil (Table 3) suggests that remimazolam may cause antegrade amnesia due to residual effects. Remimazolam itself can directly affect memory. An animal study showed that remimazolam delays the decline of memory but that the effect is transient [14]. Because there is no antagonist for propofol, it has been assumed that propofol also has residual effects just after surgery [15–17]. However, we found that memory was retained after general anesthesia with propofol, which suggests that propofol causes less antegrade amnesia than remimazolam. Since the patient sample of this study was small, however, further research is needed to address this issue.

Recovery of consciousness but no memory after general anesthesia with remimazolam can be an advantage in some clinical settings. During the recovery period from anesthesia, verbal responses are important and can help with the decision to extubate the tracheal tube; however, the experience is often unpleasant for the patient. Following sedation for a surgical procedure or during endoscopy examinations at outpatient clinics, in contrast, consciousness with no memory can be a disadvantage, as legal and ethical issues with potentially serious consequences could arise if an accident or incident occurs during this period of time.

In our study, the retention of preoperative memories was preserved in all patients after anesthesia with either remimazolam or propofol. It has been reported that midazolam (0.1 mg/kg) was associated with retrograde amnesia for a brief period regarding visual and event memory in patients undergoing caesarean delivery with spinal anesthesia [18]. The overall retrograde card recall rate was lower in the midazolam group compared with the control (no-sedatives) group (77.0 ± 13.4 vs. 87.7 ± 3.9%, *p* < 0.001), especially at 1 min before midazolam administration (58% vs. 88%, *p* < 0.001) in non-RCT setting. On the other hand, earlier studies reported that retrograde amnesia could not be induced with midazolam or diazepam [4, 12]. It has also been reported that administration of midazolam (2–10 mg) did not lead to immediate retrograde amnesia in an RCT [11]. These findings, including the present results, suggest that benzodiazepines do not induce retrograde amnesia.

### Limitations

This study was a single-center study; since it represents an exploratory trial, it should be validated by a multi-center confirmative trial. Additionally, the patient sample consisted of only female patients. However, since there are no reports of differences in memory retention between men and women, priority was given to uniformity in the degree of surgical invasion. We assessed only memory recollection (recall without showing the earlier presented item), not recognition (i.e., recognizing the item when it is subsequently shown). Recognition is more robust than recollection, but the latter still represents a very real memory trace. Additionally, the sample size was possibly inadequate to conclude that there was a difference in memory capacity. However, a post hoc power analysis for the primary endpoint yielded a value of 0.85. The analysis assumed an effect size index of 0.75 and 64 degrees of freedom and was conducted using G*Power software. The difference in the median number of posters remembered (the primary outcome) was 2.0 (2/4: 50%). In a previous report, the difference in the mean number of posters patients remembered was 1.55 (1.55/5: 30%), for a comparison between the moment immediately after anesthesia and 30 min after discharge from the post-anesthesia care unit [8]. This difference between studies could be very relevant, because the period of memory loss can be clinically dangerous. Further investigation into theses issue is therefore needed.

## Conclusions

Remimazolam restores consciousness quickly but leads to delayed recovery of memory.

## Supplementary Information

**Additional file 1.**

## Data Availability

Not applicable.
