# Supplementary figures and images for: Recovery of memory retention after anesthesia with remimazolam: an exploratory, randomized, open, propofol-controlled, single-center clinical trial
Source: JA Clin Rep. 2023 Jul 13;9:41. doi: 10.1186/s40981-023-00635-7 (PMC10338417; doi:10.1186/s40981-023-00635-7)

**Supplement 1**


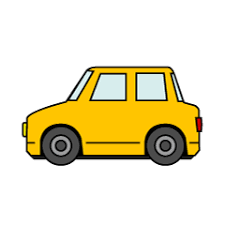

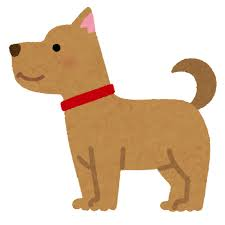

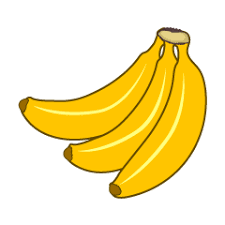

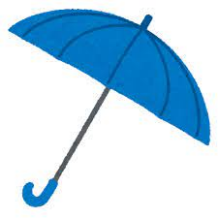

Supplement: Supplementary file 1 — Additional file 1. [file 40981_2023_635_MOESM1_ESM.docx]
